# Are anxiety disorders associated with accelerated ageing and cognitive decline? A multicenter italian study in middle aged and older patients and controls

**DOI:** 10.1192/j.eurpsy.2021.508

**Published:** 2021-08-13

**Authors:** S. Daccò, D. Caldirola, A. Alciati, A. Fagiolini, P. Brambilla, G. Perna

**Affiliations:** 1 Department Of Clinical Neurosciences, Villa San Benedetto Menni Hospital, Hermanas Hospitalarias, Albese con Cassano, Italy; 2 Mental Health, Humanitas University, Rozzano, Italy; 3 Ircc, Humanitas Clinical and Research Center, Milan, Italy; 4 Department Of Molecular And Developmental Medicine, University of Siena, Siena, Italy; 5 Pathophysiology And Transplantation, university of Milan, Milano, Italy

**Keywords:** anxiety disorders, ageing, neuropsychological performance, Cognitive decline

## Abstract

**Introduction:**

Anxiety Disorders (AnxDs) are highly prevalent in middle-aged and older individuals and are putative factors that might interfere with normal aging, by affecting cognitive functioning and neuroprogression.

**Objectives:**

This study aims to assess whether current AnxD in middle-aged and older subjects are associated with 1) lower neuropsychological performance, 2) shorter telomere length/lower plasmatic Amyloid-Beta, and 3) brain connectivity alterations, compared to subjects without lifetime psychiatric disorders (HCs).

**Methods:**

This is an ongoing multicentric cross-sectional study. We collected preliminary data on neuropsychological performance through a standardized battery, in 60 outpatients with current AnxDs (DSM-5 criteria), 24 with psychopharmacological treatments (AnxDs+) and 36 without (AnxDs-), compared to 76 HCs, all aged 50-75 years. This study was supported by Fondazione Cariplo, grant n° 2014:0664.

**Results:**

AnxDs- patients showed poorer performance in the language domain, namely in semantic fluency (p=0.04), compared to HCs. No other significant differences were found between groups. Within the patients’ group, we found that a greater burden of psychiatric disorders or medical diseases, current use of benzodiazepines, or unhealthy lifestyle had significant detrimental effects on cognition, whereas current use of antidepressants, pharmacological treatments for medical conditions, and higher levels of physical activity exhibited the opposite effects.

**Conclusions:**

We found only limited difference in cognitive performance between patients and controls. However, our preliminary results show that multiple factors influence cognitive performance in individuals with AnxDs, making these aspect important to monitor in clinical practice. So far, our results are provisional and further analyses in the final sample may provide more reliable conclusions.

